# Value of palliative surgery in perihilar cholangiocarcinoma

**DOI:** 10.1007/s00423-023-02854-z

**Published:** 2023-03-29

**Authors:** Felix Dondorf, Oliver Rohland, Aladdin Ali Deeb, Michael Ardelt, Utz Settmacher, Falk Rauchfuss

**Affiliations:** Department of General, Visceral and Vascular Surgery, Jena University Hospital, Am Klinikum 1, 07747 Jena, Germany

**Keywords:** Klatskin Tumor, Palliative care, Palliative surgery

## Abstract

**Purpose:**

The survival rate of patients with irresectable perihilar cholangiocarcinoma is remarkably poor. An essential part of palliation is treatment of obstructive cholestasis caused by the tumor. Currently, this is mainly performed endoscopically by stent or via PTBD, requiring frequent changes of the stents and limiting health-related quality of life due to the multiple hospital stays needed. The aim of this study was to evaluate surgical palliation via extrahepatic bile duct resection as an option for palliative treatment.

**Methods:**

Between 2005 and 2016, we treated 120 pCCC patients with primary palliative care. Three treatment strategies were retrospectively considered: extrahepatic bile duct resection (EBR), exploratory laparotomy (EL), and primary palliative (PP) therapy.

**Results:**

The EBR group required significantly less stenting postoperatively, and the overall morbidity was 29.4% (EBR). After the surgical procedure, fewer subsequent endoscopic treatments for stenting or PTBD were necessary in the EBR group over time. The 30-day mortality was 5.9% (EBR) and 3.4% (EL). The median overall survival averaged 570 (EBR), 392 (EL), and 247 (PP) days.

**Conclusions:**

In selected pCCC patients, palliative extrahepatic bile duct resection is a feasible option for treatment of obstructive cholestasis and should be reconsidered as a therapy option for these patients even in a palliative setting.

## Introduction

Perihilar cholangiocarcinoma (pCCC) is a rare malignant tumor that is often detected (too) late due to its nonspecific clinical presentation [1]. In the majority of cases, the first clinical sign is a painless icterus. The only option for curation is R0 resection with lymphadenectomy [2, 3]. In many cases, the tumor is already advanced [4]. If curative surgery is no longer an option, the question of available palliative therapy options is often raised. One of the main goals of palliative therapy is elimination of the obstructive cholestasis caused by the tumor. In addition to percutaneous transhepatic cholangiography and drainage (PTBD), this can be achieved by endoluminal stent therapy. Currently, stent placement and PTBD (plastic vs. metal, PTBD) are the most common treatment options [5]. Other locoregional therapies, such as photodynamic therapy, radiofrequency ablation, transarterial chemoembolization (TACE), drug-eluting bead TACE, selective intraarterial radiotherapy with 90-Y microspheres, and external beam radiation therapy, are available, but currently, no prospective, randomized controlled trials have shown a survival benefit with these therapies [6].

The main requirement for palliative therapy is to offer the patient good liveability, which in our opinion means minimizing the number of hospitalizations as much as possible. In addition to the possibility of stenting or PTBD, both of which entail regular changes, extrahepatic bile duct resection with bilioenteric anastomosis could be performed. Elimination of cholestasis is of enormous relevance not only to the patient’s comfort but also for the implementation of oncologic therapy, since biliary drainage is necessary prior to both systematic and/or radiation therapy. The aim of this study was to evaluate surgical palliation via extrahepatic bile duct resection as an option for palliative treatment.

## Methods

Between 2005 and 2016, 225 pCCC patients were treated in our center, of which 105 patients were treated curatively (87 with major liver resection and 18 with transplantation), and 120 patients were treated palliatively. The palliative treated population was divided into three treatment groups: 17 patients received palliative extrahepatic bile duct resection (EBR), 59 patients underwent exploratory laparotomy (EL) with initial curative intention, and 44 patients were treated with primary palliative (PP) therapy (without surgical treatment) after diagnosis. The primary, palliative therapy consisted primarily of endoscopic biliary decompression with the aim of subsequent systematic chemotherapy, if the overall morbidity of the patients permitted this. Alternatively, the patients were given a “best supportive care” therapy concept. The decision among the therapeutic options was made at the interdisciplinary hepatopancreatobiliary tumor conference of our institution and/or intraoperatively depending on the findings regarding tumor expansion.

Basic patient parameters, perioperative data, and postoperative follow-up data were collected; three groups (EBR, EL, PP) were compared with each other in the sense of a quantitative intervention study. Patient-related data of the samples were collected. Statistical analysis of the data was performed using the statistical software SPSS (IBM, Armonk, NY, USA) and Microsoft Excel (Microsoft Corporation, Redmond, USA). Primarily, a single factor analysis of variance was performed; the significance level was *p* < 0.05.

Permission for this study was granted by the institutional review board and approved as part of patient care. The additional approval number is Reg. nr.: 2021–2327-data.

## Results

In the EBR group, 5 patients (29.5%) were female and 12 patients (70.5%) were male with a mean age of 67 ± 8.4 years; in the EL group, 25 patients (42.4%) were female and 34 patients (57.6) were male with a mean age of 63 ± 9.7 years; and in the PP groups, 17 patients (38.6%) were female and 27 patients (61.4%) were male with a mean age of 70 ± 11, 3 years.

Extrahepatic bile duct resection was performed in patients who could not undergo radical and curative-intended surgery due to metastases (*n* = 4), the liver parenchyma quality (liver cirrhosis or fibrosis) or an inadequate FRL (*n* = 13); thus, we attempted to remove the main tumor mass as part of surgery. The procedure was performed as a Roux-en-Y choledochojejunostomy, the biliodigestive anastomoses were stented with a T-drain. Patients in the exploration group underwent laparotomy, and the curative-intended surgery was not completed due to the reasons presented in Table 1. Patients who were already classified as nonresectable based on diagnostic investigations (CT, MR, MRCP, PET, ERCP) or who could not be treated surgically due to comorbidities were assigned to the primary palliative therapy regimen. Regarding biliary decompression (both via stent and PTBD), the data shown in Table 2 were obtained. Remarkably, in the EBR group, only 17.6% of the patients had to be treated with new drainage postoperatively. In the EL and PP groups, > 90% of the patients were treated with new bile duct drainage (1 patient in the PP group died prior to the change). Statistical analysis comparing preoperative and postoperative values showed that patients in the EBR group required significantly less stenting postoperatively (*p* < 0.001).

**Table 1:** List of reasons for which exploratory laparotomy was terminated in the 59 patients in the group. The number of cases is listed, and the calculated percentages are given in parentheses

Cause of exploratory laparotomy termination	*n* (%)
Peritoneal carcinomatosis	18 (30,5)
Liver cirrhosis	8 (13,6)
Tumor invasion of other organs	11 (18,6)
(Arterial) vessel infiltration	12 (20,4)
Metastasis (in FRL or distant in the abdominal cavity) unknown prior laparotomy	10 (16,9)

**Table 2:** Presentation of preoperative and postoperative interventions (ERCP + stent vs. PTBD) divided by treatment group

	EBR	EL	PP
	*n* = 17 (%)	*n* = 59 (%)	*n* = 44 (%)
Intervention pre- and postoperative			
PTBD preoperative	0	4 (6,8)	x
PTBD postoperative	3 (17,6)	9 (15,3)	x
Stent preoperative	15 (88,2)	56 (94,9)	x
Stent postoperative	0	55 (93,2)	x
Stent	x	x	43 (97,7)
Bismuth-Corlette classification			
I	9 (52,9)	1 (1,7)	0
II	5 (29,4)	6 (10,2)	3 (6,8)
IIIa	1 (5,9)	6 (10,2)	5 (11,4)
IIIb	0	8 (13,6)	5 (11,4)
IV	2 (11,8)	31 (52,6)	28 (63,7)
n/a	0	7 (11,9)	3 (6,8)
Complications			
Over all complication	5 (29,4)	8 (13,6)	5 (11,4)
Relaparotomy	1 (5,9)	4 (6,8)	x
Bile leakage	1 (5,9)	4 (6,8)	x
Biliom	1 (5,9)	6 (10,2)	x
Clavien-Dindo classification (major complication > IIIa)			
IIIa	2 (11,8)	8 (13,6)	x
IIIb	1 (5,9)	4 (6,8)	x
IVa	1 (5,9)	1 (1,7)	x
IVb	0	3 (5,1)	x
V	0	2 (3,4)	x
Duration of diagnosis/stay			
Time from diagnosis to surgery	70 ± 131,3	86 ± 137,9	x
Hospital stay	24 ± 11	15 ± 7,8	33 ± 33,1
ICU stay	1,2 ± 0,4	1,7 ± 0,5	1,8 ± 0,4
Survival			
30 day mortality	1 (5,9)	2 (3,4)	x
Survival overall (in d)	570,7 ± 456,8	392,9 ± 409,2	247,7 ± 251,3
Survival postoperative (in d)	505,9 ± 422,8	319,7 ± 407,5	x

The number of cases is given, the percentages are shown in parentheses and the mean values + standard deviation are presented. Division of the individual treatment paths according to the Bismuth–Corlette classification. Complications and major complications (CD > IIIa) broken down by treatment group. Diagnosis to surgery interval, length of hospital stay and ICU stay in days. Mean values + standard deviation are shown, divided by treatment group. 30-day mortality, median overall survival and postoperative survival in days, divided by treatment

The distribution of Klatskin tumors according to the Bismuth–Corlette classification is shown in Table 2. The TNM classification of the histological specimens, when any could be obtained, is listed in Table 3. In the EL and PP groups, no specimens were usually obtained (see Table 3). A total of 7 patients had an R + (R1/R2) resection. Of these, the distal margin was positive in 2 patients, the proximal margin was positive in 2 patients, and both margins were positive in 3 patients. There was no rehospitalization in the distal margin and in both positive margin. Only in the group of patients with proximal positive margin, one patient had to be rehospitalized.

**Table 3:** Tumor formula of the histological specimens divided by treatment group

		0	1	2	3	4	n/a
T	EBR	x	x	9 (52,9)	5 (29,4)	1 (5,9)	2 (11,8)
		0	1	n/a			
N	EBR	8 (47,1)	3 (17,6)	6 (35,3)			
		0	1	n/a			
M	EBR	11 (64,7)	x	6 (35,3)			
	EL	15 (25,4)	7 (11,9)	37 (62,7)			
		0	1	n/a			
L	EBR	9 (52,9)	5 (29,4)	3 (17,6)			
	EL	1 (1,7)	1 (1,7)	57 (96,6)			
		0	1	2	n/a		
V	EBR	8 (47,1)	4 (23,5)	2 (11,8)	3 (17,6)		
	EL	2 (3,4)	x	x	57 (96,6)		
		0	1	n/a			
PN	EBR	1 (5,9)	6 (35,3)	10 (58,8)			
	EL	x	2 (3,4)	57 (96,6)			
		1	2	3	n/a		
G	EBR	2 (11,8)	5 (29,4)	7 (41,2)	3 (17,6)		
	EL	1 (1,7)	4 (6,8)	3 (5,1)	51 (86,4)		
	PP	1 (2,3)	x	2 (4,5)	41 (93,2)		
		0	1	2	n/a		
R	EBR	8 (47,1)	2 (11,8)	3 (17,6)	4 (23,5)		
	EL	x	x	6 (10,2)	53 (89,8)		

Overall morbidity was 29.4% (5 patients) in the EBR group and 13.8% (8 patients) in the EL group. The distribution of major complications according to Clavien–Dindo (CD > IIIa) classification is shown in Table 2. A total of 5.9% (1 patients) of the EBR group and 6.8% (4 patients) of the EL group underwent relaparotomy due to complications.

After completion of the surgical procedure, only for 3 patients endoscopic treatments with stenting or PTBD were necessary in the EBR group. In the EL group, 76.3% underwent stent replacement, 11.9% subsequently underwent metal mesh stenting, and 3.4% underwent PTBD. In the PP group, 77.3% received a stent change, 18.2% a metal mesh stent, and 2.3% a PTBD. In the univariate variance analysis, patients in the EBR group required significantly fewer palliative interventions than patients in the EL and PP groups (*p* < 0.001). Related to all possible causes and problems only 41.17% (7/17) had to be hospitalized again after EBR group during the follow-up.

Among all groups, 20.8% (25 patients) received chemotherapy: 13 patients received a combination of gemcitabine + cisplatin, 11 patients received gemcitabine monotherapy, and 1 patient received cisplatin monotherapy. The remaining patients did not receive chemotherapy.

The time between diagnosis and surgical intervention averaged 70 days in the EBR group and 86 days in the EL group. The hospitalization time was 24 days in the EBR group and 15 days in the EL group (significantly different EL vs. EBR p = 0.012), with a mean ICU stay of 1.2 days (EBR) and 1.7 days (EL). The hospitalization time for the primary palliative patients was 32 days (significantly different EL vs. PP *p* = 0.004), with a mean ICU stay of 1.8 days (Table 2). The 30-day mortality was 5.9% in the EBR group and 3.4% in the EL group (significantly different EBR/EL vs. PP *p* < 0.001). OS averaged 570 days in the EBR group, 392 days in the EL group, and 247 days in the primary palliative group (Table 2). Regarding the extent of tumor locally in EBR group, we performed survival in subgroups by R status and demonstrated, that R0 resected patients had longer OS (713 ± 519) than R1 (487 ± 303) and R2 resected (166 ± 170), although not significantly due to single group size.

**Fig. 1:**
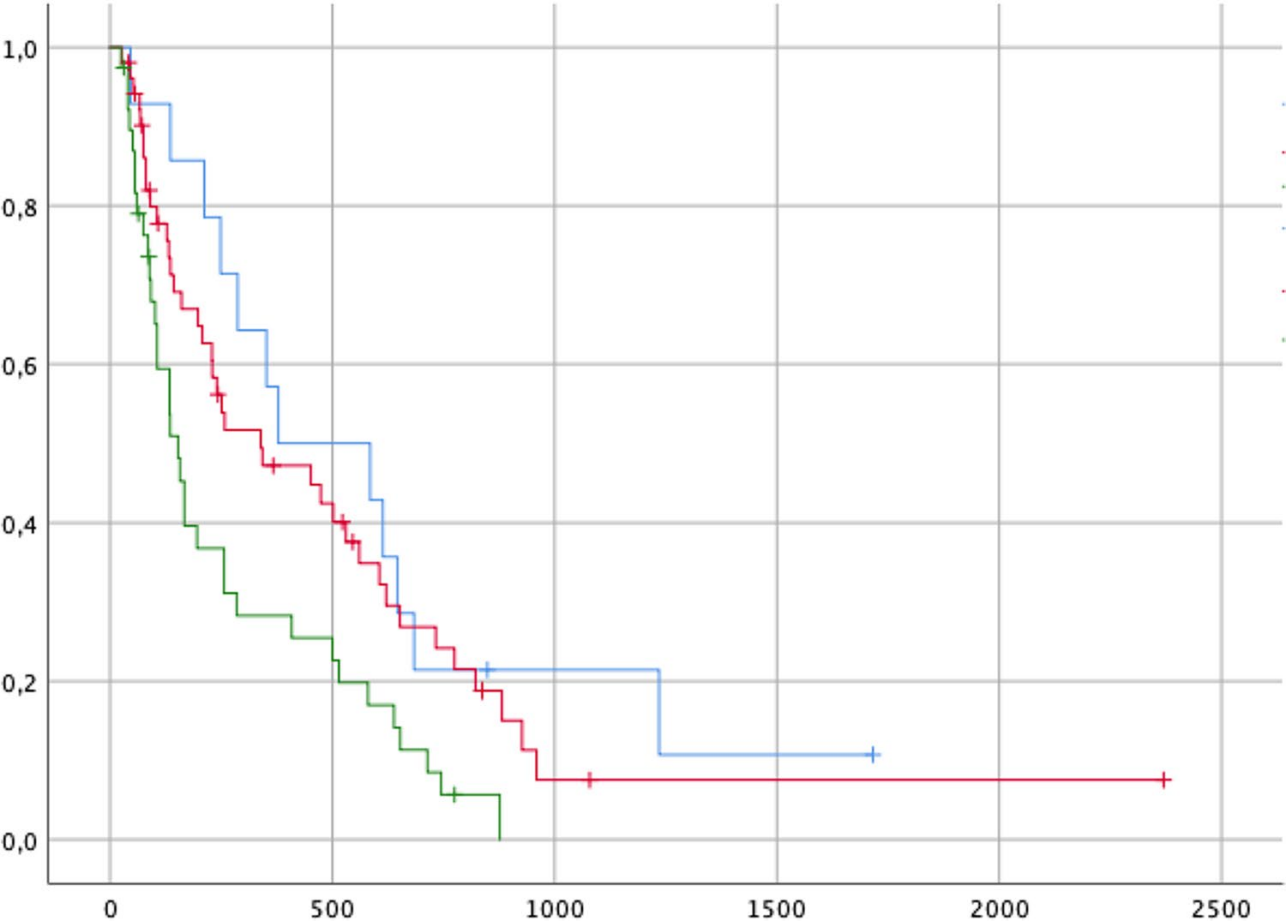
Kaplan–Meier survival curves divided by therapy variant (blue: EBR, red: EL, green: PP)

Evaluation of the survival curves showed a nonsignificant difference in the treatment variant, see Fig. 1. Univariate analysis of variance showed a significant difference in OS between the EBR and PP groups (*p* = 0.015).

## Discussion

It is well known that pCCC patients have poor outcomes, especially when therapy is purely palliative and biliary tree obstruction has important consequences, mainly for the patient’s health-related quality of life (HRQoL), morbidity, and overall mortality [7]. Therefore, Vasilieva et al. concluded that it is absolutely necessary that a diagnostic approach be adopted to ensure timely confirmation of the disease to maximize the patient’s benefit from therapeutic intervention in terms of overall survival and HRQoL [8].

Currently, endoscopic procedures with stenting or external drainage using PTBD are the leading treatments. However, these procedures also have limitations [9]. For example, as early as 1998, Jarnagin et al. demonstrated that cholangitis and recurrent obstruction are the principal complications of palliative biliary drainage, require further intervention and must be minimized to preserve HRQoL and their findings are still relevant today [10]. Based on this, we investigated which palliative therapeutic options are available in addition to stenting alone. We were able to show that palliative EBR is also an option for pCCC patients compared with stenting or purely palliative therapy.

A major issue that is always discussed (not only in palliative pCCC patients) is the indication for as well as the mode of biliary drainage prior to surgical intervention. This discussion remains controversial [6, 11–13]. Thus, the present study clearly shows that most patients are stented before surgery or at the time of treatment initiation (> 90% of patients, regardless of the treatment arm). Most patients present to the hepatobiliary center after initial treatment at smaller hospitals, which not only complicates the diagnosis but also alters the operative/postoperative outcome.

Van Keulen et al. found that treatment at a specialized center resulted in a higher resection rate [14]. Therefore, to ensure that the time between diagnosis and surgery is as short as possible, we recommend that patients diagnosed with pCCC are rapidly presented to a hepatobiliary center. The data collected here show a median time between diagnosis and surgery of between 70 days (EBR) and 86 days (EL), which in our opinion is too long.

The present work further shows that the long-term survival of pCCC patients who cannot undergo curative resection is significantly reduced, regardless of which treatment option is applied. Goenka et al. showed that survival is almost always less than 1 year for patients without a feasible resection option [15]. Additionally, the data reported by van Keulen et al. show that patients who received systemic therapy or best supportive care had negligible 5-year survival rates of 1.8% and 1.6% [14]. In view of the significant morbidity and mortality associated with recurrent cholangitis, careful optimization of bile duct drainage is critical for improving survival in these terminally ill patients [16].

Patients who underwent extrahepatic bile duct resection showed a significant survival advantage compared to patients who underwent primary palliative therapy, which was also observed by Hu et al., who noted that palliative surgery resulted in a relatively better outcome compared with patients who did not accept any treatment [17].

Kissenkoetter et al. confirmed that patients with pCCC who underwent R1 resection had a significant survival advantage compared to patients who received palliative therapy; therefore, R1 resection is a very effective palliation [18].

This fact favors an attempt to resect patients palliatively if this seems possible and if the patient is not eligible for curative resection (with major liver resection). Additionally, the low 30-day mortality of 5.9% in the EBR group vs. 3.4% in the EL group fits the current literature [17] and suggests that patients should receive surgical treatment (curative resection, EBR, or EL) whenever possible. Geller et al. also noted that the overall survival of pCCC patients remains poor, regardless of the palliation method used, and thus, surgery should be offered to all patients whenever possible to provide any chance for a cure [19].

**Fig. 2:**
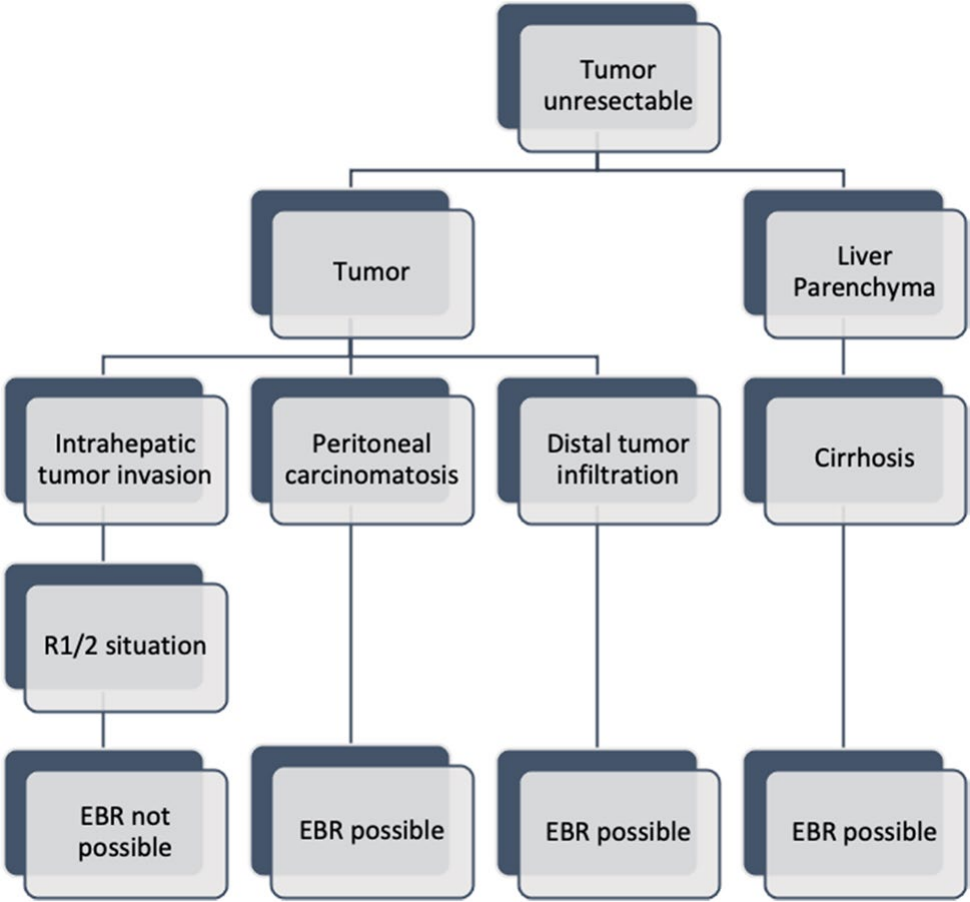
Proposal for the decision process regarding whether an EBR is possible

In our center, we always consider surgical treatment, even if only an exploratory laparotomy is performed with an attempt at resection. It is known that 10 to 45% of patients with pCCC considered resectable are found to have unresectable tumors during explorative laparotomy [6]. If the disease is not considered curable, EBR may be attempted to improve liveability. We are aware that palliative surgery in pCCC has not yet been sufficiently illuminated scientifically, which is also due to the small number of cases in the individual papers, but the following proposal for the decision process (Fig. 2) should be used when deciding on EBR. Of course, the combination of tumor and parenchyma as well as the general condition of the patient (age, BMI, comorbidities) must always be considered and a decision must be made depending on the combination of factors.

Across all groups, 20% of patients received chemotherapy. This is consistent with the current literature, as van Keulen et al. also found, that the proportion of patients who receive chemotherapy seems low, which might be caused by the reason that these patients have often liver dysfunction and hyperbilirubinemia[14].

Blechacz et al. considered that there are no prospective, large, randomized, controlled trials that have yet shown a survival benefit from either neoadjuvant or adjuvant treatments [6, 20]. This circumstance also supports the need for a palliative surgical intervention, if this seems possible considering the individual patient, to at least improve HRQoL during “best supportive care”. Another point that also supports the use of EBR or EL under palliative conditions is, that these procedures can allow samples to be obtained for histology in preparation for systemic therapy, which is sometimes difficult with brush cytology, as studies have demonstrated, that standard cytology brushings are negative in more than 50% of cases [21]. The need for histological examination was also discussed by Fong et al., who postulated that for patients undergoing systemic chemotherapy or radiation therapy, tissue diagnosis is needed prior to treatment initiation [21, 22].

The retrospective character as well as the small number of EBR patients cannot be argued away and must be acknowledged as a major limitation of the work. Nevertheless, it became clear that EBR (even in the small collective) is feasible as an alternative for a selected patient population. Assuming now, that the majority of patients undergo exploration with curative intent, EBR (as a minimal surgical variant) is a palliative option compared to endoscopic palliation. Of course, it should also be noted that overall survival is reduced in patients treated primarily with palliative therapy and thus, there is a selection disadvantage here. If we consider the two surgical therapy arms, it is noticeable that type IV Klatskin tumors (according to the Bismuth–Corlette classification) were mainly found in the EL group compared to the EBR group, which also shifts the selection.

As shown by Geller et al., despite all the recent innovations in interventional endoscopy and radiology, palliative therapy for patients with advanced hilar malignant obstruction is still suboptimal [19]. Ashat et al. reported that draining more than 50% of the liver volume is an important predictor of treatment effectiveness [23].

Therefore, the present work demonstrates that extrahepatic biliary resection in a palliative setting for pCCC can be a therapeutic option compared with the “best supportive care” approach (with regular biliary decompression) and can provide patients with better HRQoL (fewer palliative interventional procedures and a reduced number of hospitalizations). Mihalache et al. also demonstrated that quality of life was improved by therapy, either radical or palliative, because endoscopic palliative treatment allowed faster community reintegration compared with palliative surgery, which was followed by improvement only after a certain period of time [24]. Similarly, Sotiropoulos et al. noted that hepatojejunostomy was a potentially successful method [25].

## Conclusion

pCCC patients should always be treated with curative intent. If this is not possible, we show that palliative EBR has advantages compared to purely palliative therapy. Therefore, in selected palliative pCCC patients, we believe extrahepatic bile duct resection, with the creation of a bilioenteric anastomosis to eliminate obstructive cholestasis, should also be considered as an option for palliation, which would result in significantly fewer interventions for patients postoperatively and thus improve HRQoL for patients in the palliative setting. However, future work should prospectively examine this recommendation.
